# Causality of genetically determined blood metabolites on inflammatory bowel disease: a two-sample Mendelian randomization study

**DOI:** 10.1038/s41598-024-67376-0

**Published:** 2024-07-16

**Authors:** Xiongquan Long, Yuyang Zhang, Mingzhu Liu, Zihao Liu, Lvzhou Xia, Xiaoping Xu, Minghao Wu

**Affiliations:** 1 Department of Gastroenterology, The First Affiliated Hospital of Hunan Normal University, Hunan Provincial People’s Hospital, Changsha, 410005 Hunan China; 2 Central Laboratory of Hunan Provincial People’s Hospital, The First Affiliated Hospital of Hunan Normal University, Changsha, 410005 Hunan China; 3 Department of Endoscopic Diagnosis and Treatment Center, Hunan Cancer Hospital, The Affiliated Cancer Hospital of Xiangya School of Medicine, Central South University, Changsha, 410005 Hunan China

**Keywords:** Computational biology and bioinformatics, Genetics, Immunology, Gastroenterology

## Abstract

Inflammatory bowel disease (IBD) is a chronic and recurrent inflammatory disease of the gastrointestinal tract, including two subtypes: Crohn’s disease (CD) and ulcerative colitis (UC). Metabolic disorders are important factors in the development of IBD. However, the evidence for the causal relationship between blood metabolites and IBD remains limited. A two-sample MR analysis was applied to evaluate relationships between 486 blood metabolites and IBD. The inverse variance weighted method was chosen as the primary MR analysis method. False discovery rate correction was used to control for false positives in multiple testing. Following complementary and sensitivity analyses were conducted using methods such as weight median, MR-egger, weighted mode, simple mode, Cochran Q test, and MR-PRESSO. Moreover, we performed replication, meta-analysis, Steiger test, and linkage disequilibrium score regression to enhance the robustness of the results. Additionally, we performed metabolic pathway analysis to identify potential metabolic pathways. As a result, we identified four significant causal associations between four blood metabolites and two IBD subtypes. Specifically, one metabolite was identified as being associated with the development of CD (mannose: odds ratio (OR) = 0.19, 95% confidence interval (CI) 0.08–0.43, *P* = 8.54 × 10^–5^). Three metabolites were identified as being associated with the development of UC (arachidonate (20:4n6): OR = 0.18, 95% CI 0.11–0.30, *P* = 2.09 × 10^–11^; 1, 5-anhydroglucitol: OR = 2.21, 95% CI 1.47–3.34, *P* = 1.50 × 10^–4^; 2-stearoylglycerophosphocholine: OR = 2.66, 95% CI 1.53–4.63, *P* = 5.30 × 10^–4^). The findings of our study suggested that the identified metabolites and metabolic pathways can be considered as useful circulating metabolic biomarkers for the screening and prevention of IBD in clinical practice, as well as candidate molecules for future mechanism exploration and drug target selection.

## Introduction

Inflammatory bowel disease (IBD) is a chronic and recurrent inflammatory disease of the gastrointestinal tract, including two subtypes: Crohn's disease (CD) and ulcerative colitis (UC). Worldwide, the prevalence of IBD is gradually increasing, especially in newly industrialized countries (China and India), with a prevalence of 0.5% in 2010, 0.75% in 2020, and projected to reach 1% in 2030. The increasing prevalence of the disease has led to an increase in hospitalization rates, which adds to the economic burden on health-care systems around the world^1,2^. Patients with IBD usually present clinically with chronic recurrent diarrhea, abdominal pain, and mucous bloody stools, and may develop serious complications such as intestinal perforation, intestinal obstruction, and anal fistulas^3,4^. However, clinically, the diagnosis of IBD lacks a gold standard and often requires a combination of endoscopic, histologic, and laboratory investigations, which makes it difficult to differentiate it from other nonspecific enterocolitis^5^. Furthermore, despite significant advances in the pharmacologic treatment of IBD with the introduction of novel biologic and small molecule therapies, patients pay a high price both financially and physically. Notably, these therapies do not cure IBD and may have life-threatening side effects. A considerable number of patients eventually require surgery after the treatment^6–8^. It highlights the need for new biodiagnostic markers and therapeutic strategies for IBD. However, the etiology and pathogenesis of IBD have not been fully elucidated. It is generally recognized that IBD is a persistent excessive immuno-inflammatory response resulting from the interaction of genetic, microbial and environmental factors^9,10^.

In recent years, the emergence of metabolomics as a component of systems biology has provided a new way to investigate the pathogenesis of diseases. There are increasing evidences that metabolic disorders are closely related to the development of IBD. For example, Scoville et al. found that 173 metabolites were significantly altered (27 increased and 146 decreased) by metabolomic analysis of serum collected from IBD patients and healthy controls. Most of the alterations occurred in lipids, amino acids, and energy-related metabolites^11^. Di'Narzo et al. found that serum from IBD patients was enriched in fibrinogen cleavage peptides as well as xanthine and tryptophan metabolites compared to healthy controls. Whereas fibrinogen cleavage polypeptide and diacylglycerols were significantly differentially expressed in CD versus UC patients^12^. However, the detailed pathophysiologic mechanisms of blood metabolites in IBD remain unclear. Moreover, due to limitations in sample size, confounding factors, and ethical issues, there are no prospective studies or randomized controlled trials (RCTs) to confirm their causal relationship. Therefore, there is an urgent need for a comprehensive and in-depth analysis of the causal role of blood metabolites in the pathogenesis of IBD.

With the development of high-throughput technologies, it is now possible to measure hundreds of circulating metabolites and genotype them in parallel in large populations^13^. Genome-wide association studies (GWAS) can provide molecular insight into the complex interplay of genetic factors and environmental factors in disease pathogenesis. To date, a considerable number of single nucleotide polymorphisms (SNPs) have been found to be strongly correlated with blood metabolites. However, there are still significant barriers to translating these genetic findings into the biological mechanisms of IBD development, requiring in-depth analyses to reveal the causal interactions of blood metabolites on IBD susceptibility.

Mendelian randomization (MR) analysis is a new and powerful epidemiological tool that has recently gained widespread use in disease etiology studies. MR assesses the causal impact of exposure on outcomes by selecting exposure-related SNPs as instrumental variables (IVs). In detail, this IVs alternative approach mimics the design of RCTs because SNPs are randomly assigned to offspring at conception, which largely avoids the effects of confounding factors^14,15^. Recently, GWAS was extended to metabolic phenotyping, producing a genetically determined metabolites (GDMs) atlas^16^. Inspired by the dataset of GDMs, we implemented a two-sample MR approach to (1) assess the causal effects of human blood metabolites on IBD; (2) explore whether there are common metabolites with causal effects on CD and UC; and (3) identify potential metabolic pathways that may contribute to the development of the IBD.

## Materials and methods

### Study design

**Figure 1:**
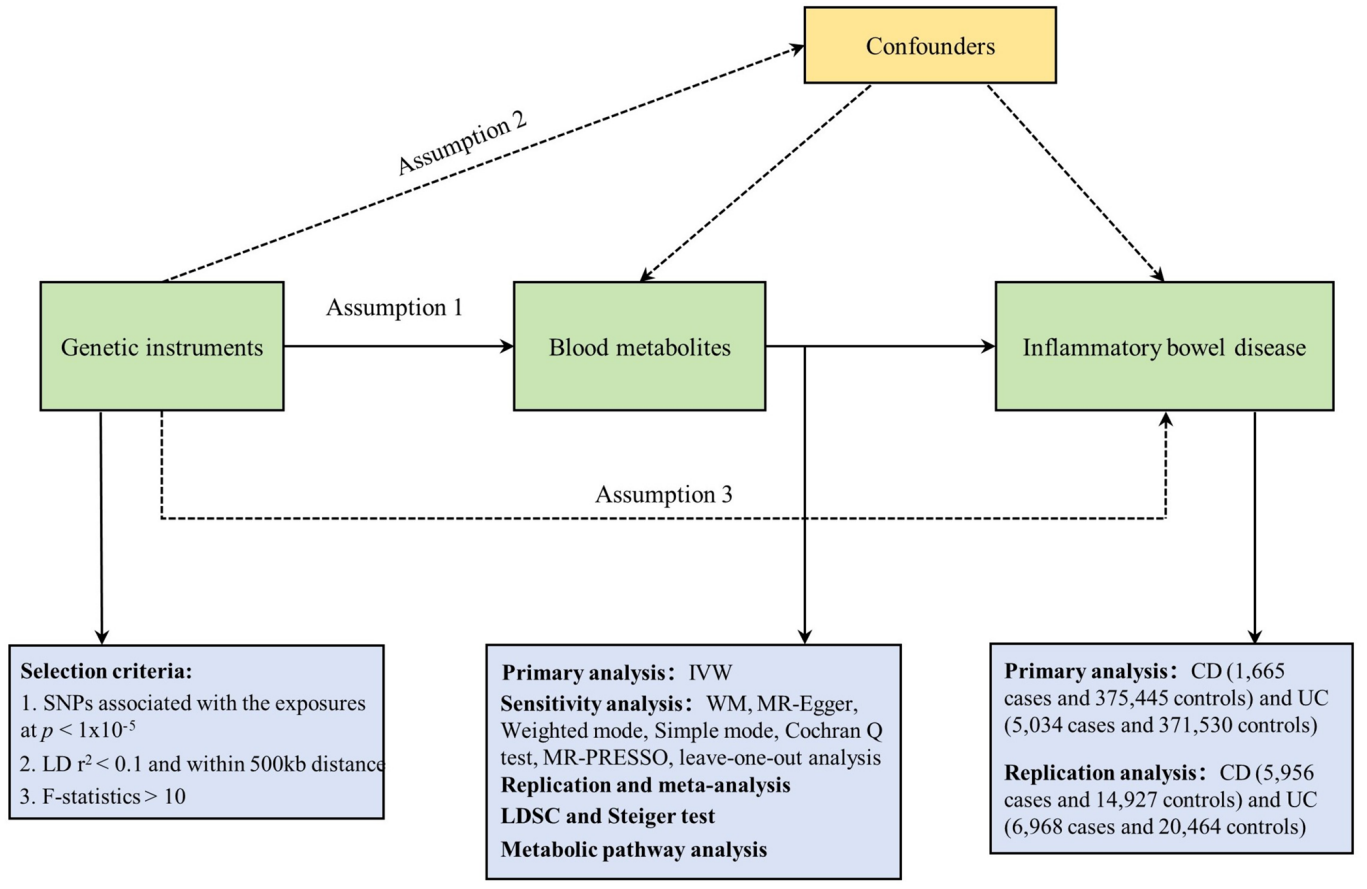
Overview of this Mendelian randomization (MR) analysis. IVW, inverse variance weighted; LD, linkage disequilibrium; LDSC, linkage disequilibrium score regression; SNPs, single nucleotide polymorphisms; WM, weighted median.

We systematically assessed the causal relationship between human blood metabolites and the risk of IBD by using a two-sample MR design. Compelling MR studies should meet 3 basic assumptions: (1) the genetic instruments should be directly associated with exposure (i.e., metabolites in this study); (2) the genetic instruments should be independent of outcome (i.e., IBD in this study) and independent of any known or unknown confounders; (3) the effect of IVs on outcome is mediated only by the exposure of interest. Genetic information for metabolites and IBD were obtained from separate GWAS datasets to avoid sample overlap. The study methods were compliant with the STROBE-MR checklist^15^. An overview of this MR study is shown in Fig. 1.

### GWAS data for blood metabolites and IBD

The genome-wide association summary datasets of 486 metabolites associated with human genetic variation were obtained from the study by Shin et al.^16^. This is the most comprehensive analysis of human metabolites to date, with complete summary statistics available through the Metabolomics GWAS server (http://metabolomics.helmholtz-muenchen.de/gwas/). This GWAS analysis included 7824 adults and approximately 2.1 million SNPs from two European cohorts. In more detail, 1768 of these participants were from the KORA F4 study in Germany, and 6056 were from the UK Twin Study. Of the 486 metabolites, 309 were known metabolites, which can be grouped into eight categories: cofactors and vitamins, energy, amino acids, carbohydrates, lipids, nucleotides, peptides, and xenobiotic metabolisms, as documented in the Kyoto Encyclopedia of Genes and Genomes (KEGG) database. Another 177 were unknown metabolites whose chemical identity had not yet been determined.

To minimize bias due to racial differences and sample overlap, GWAS data for IBD from the FinnGen database (https://www.finngen.fi/en, R8 released in 2023) were used for the primary analysis^17^. In this data, UC and CD were strictly diagnosed by ICD codes and required KELA and at least 2 HDR. Specific information of participants, genotype platforms, data survey methods are available at the FinnGen website. The dataset used consisted of 1,665 patients with CD and 375,445 controls (phenocode: K11_CD_STRICT2), and 5,034 patients with UC and 371,530 controls (phenocode: K11_UC_STRICT2).

### Selection of IVs

In order to fulfill the 3 basic assumptions of the MR study, we identified IVs associated with blood metabolites from multiple perspectives through rigorous screening conditions. First, considering the limited number of metabolite-related SNPs and referring to some previous studies, we set the significance threshold at p < 1 × 10^–5^ and the linkage disequilibrium (LD) r^2^< 0.1 within 500 kilobase (kb)^18–20^. Second, to eliminate the bias caused by poor quality IVs, we calculated the R2 and F-statistics for each SNP (the formulas are shown in Supplementary Table S1). In general, F-statistics < 10 were considered weak IVs and discarded^21,22^. Third, we extracted SNPs associated with metabolism from the results and discarded SNPs strongly associated with the results (p < 1 × 10^–5^). Fourth, we further harmonized SNPs for exposure and outcome and eliminated SNPs with palindromic effects and allelic incongruence. Finally, to avoid the effect of confounders, PhenoScanner V2 (http://www.phenoscanner.medschl.cam.ac.uk/) was used to test the assumption that IVs are independent of confounders by analyzing genome-wide significant relationships (P < 1 × 10^–5^). Through this process, we eliminated SNPs that were associated with known risk factors for IBD, such as depression, being a worrier, smoking, taking contraceptives, having sclerosing cholangitis^23–26^.

### MR analysis and statistical analysis

The causal associations between blood metabolites and IBD for this MR analysis were primarily estimated using the inverse variance weighted (IVW) method. If there is no heterogeneity, the fixed-effect IVW method is used, and vice versa with random-effect. It is noteworthy that the IVW method can provide a consistent assessment of exposure causality when each variable satisfies all three assumptions of a valid instrumental variable^27,28^. To improve the stability and robustness of the results, we used four additional methods as complementary analyses to further evaluate the metabolite. The MR-Egger method can detect violations of the IVs assumptions and provide estimates of effects that are not affected by these violations^29^. The weighted median method is more tolerant of invalid IVs and produces plausible estimates when more than half of the weights correspond to valid IVs^30^. Moreover, simple mode and weighted mode methods were also applied as the complementary analyses^31^. In addition, we performed the following sensitivity analyses: (1) Cochran Q test was used to detect potential violations of the assumption by the heterogeneity of the association between individual IVs. Cochran Q test-derived *p* < 0.05 was considered as heterogeneity of the results^32^; (2) MR-Egger was utilized to estimate horizontal pleiotropy based on its intercept to ensure that genetic variants were independently associated with exposure and outcome^33^; (3) MR-PRESSO was applied to detect outliers and correct the horizontal pleiotropy^34^; (4) we performed leave-one-out analysis to assess whether the results were influenced significantly by a single SNP^35^. Statistical analyses were performed with R4.3.1 software, and MR analyses were performed with the MendelianRandomization package software package. MR-PRESSO was performed using the MRPRESSO package. False discovery rate (FDR) correction was used to control for false positives in multiple testing. According to previous studies, A statistically significant association was considered if the estimated causal effect of a given metabolite had a FDR < 0.1^36,37^.

As a result, we rigorously screened blood metabolites potentially causally associated with IBD by multiple criteria; (1) significant p-value (FDR_ivw_ < 0.1) for primary analyses; (2) consistency in direction and magnitude of the five MR methods; (3) no heterogeneity or horizontal pleiotropy in the MR estimates; (4) MR estimates are not heavily confounded by individual SNPs.

### Replication and meta-analysis

To fully assess the robustness of the candidate metabolites identified based on the above criteria, we replicated the IVW analysis in an additional IBD cohort from The International Inflammatory Bowel Disease Genetics Consortium. The cohort data are available at the IEU OpenGWAS database (https://gwas.mrcieu.ac.uk/). In this cohort, the CD group (GWAS ID: ieu-a-30) consisted of 5,956 European ancestry cases and 14,927 European ancestry controls, while the UC group (GWAS ID: ieu-a-32) consisted of 6,968 European ancestry cases and 20,464 European ancestry controls. The results of the meta-analysis of the two MR analyses allowed us to finally identify the blood metabolites causally associated with IBD.

### Genetic correlation and direction validation

Previous studies have shown that MR results may have false positives due to genetic correlations between traits^38^. Although SNPs associated with IBD were excluded in the selection of IVs, combinations of SNPs not significantly associated with IBD may also contribute to the genetic risk of IBD. Linkage disequilibrium score regression (LDSC) can be used to estimate co-inheritance by performing chi-square statistics for two traits based on SNP. Therefore, genetic associations between identified metabolites and IBD were assessed by LDSC to determine whether causality is confounded by shared genetic architecture. In addition, we used the Steiger test to confirm whether the observed causalities were biased due to reversed causation. When a combination of SNPs was found to have no genetic risk for IBD compared to blood metabolites, the results indicated no bias in causal inference (Steiger *p* < 0.05)^39^.

### Metabolic pathway analysis

Metabolic pathways were analyzed via the web-based MetaboAnalyst 5.0 (https://www.metaboanalyst.ca/)^40^. The functional pathway analyses module was used to identify potential metabolite pathways that may be relevant to the biological processes of IBD. The Kyoto Encyclopedia of Genes and Genomes (KEGG) database and the Small Molecule Pathway Database (SMPDB) were used in this study. Referring to previous studies, the significance level for pathway analysis was set at 0.10^41,42^.

## Results

### Selection of IVs

Of the 486 metabolites, 3–494 independent SNP component instrumental variables were collected in the CD group. Whereas in the UC group, 3–493 independent SNP component instrumental variables were collected. The F-statistics of the metabolite-related SNPs were all greater than the empirical threshold of 10, which indicated that all SNPs were sufficiently effective. Detailed data of IVs are shown in Supplementary Table S2 and S3.

### Primary analysis

**Figure 2:**
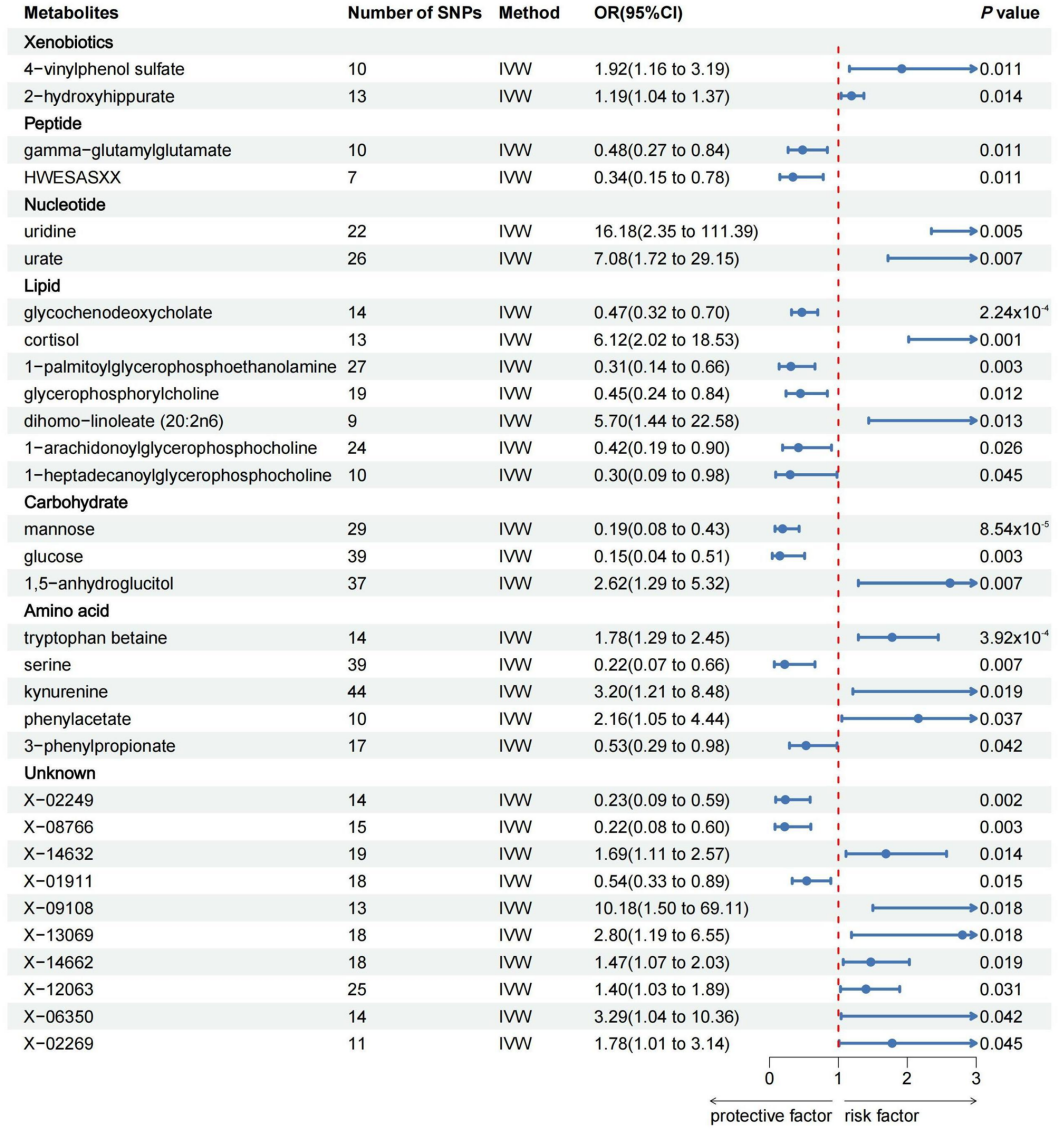
Mendelian randomization results for blood metabolites and the risks of CD based on the inverse variance weighted (IVW) method.

As shown in Fig. 2, 31 metabolites associated with CD were detected by the IVW method (*p* < 0.05), including 7 lipids, 5 amino acids, 3 carbohydrates, 2 peptides, 2 nucleotides, 2 xenobiotics and 10 unknown metabolites. Among the known metabolites, 10 metabolites were positively correlated with CD and remaining 11 metabolites were associated with a reduced risk of CD. However, after FDR correction, only glycochenodeoxycholate (FDR_ivw_ = 0.054), mannose (FDR_ivw_ = 0.041), and tryptophan betaine (FDR_ivw_ = 0.063) remained significant.

**Figure 3:**
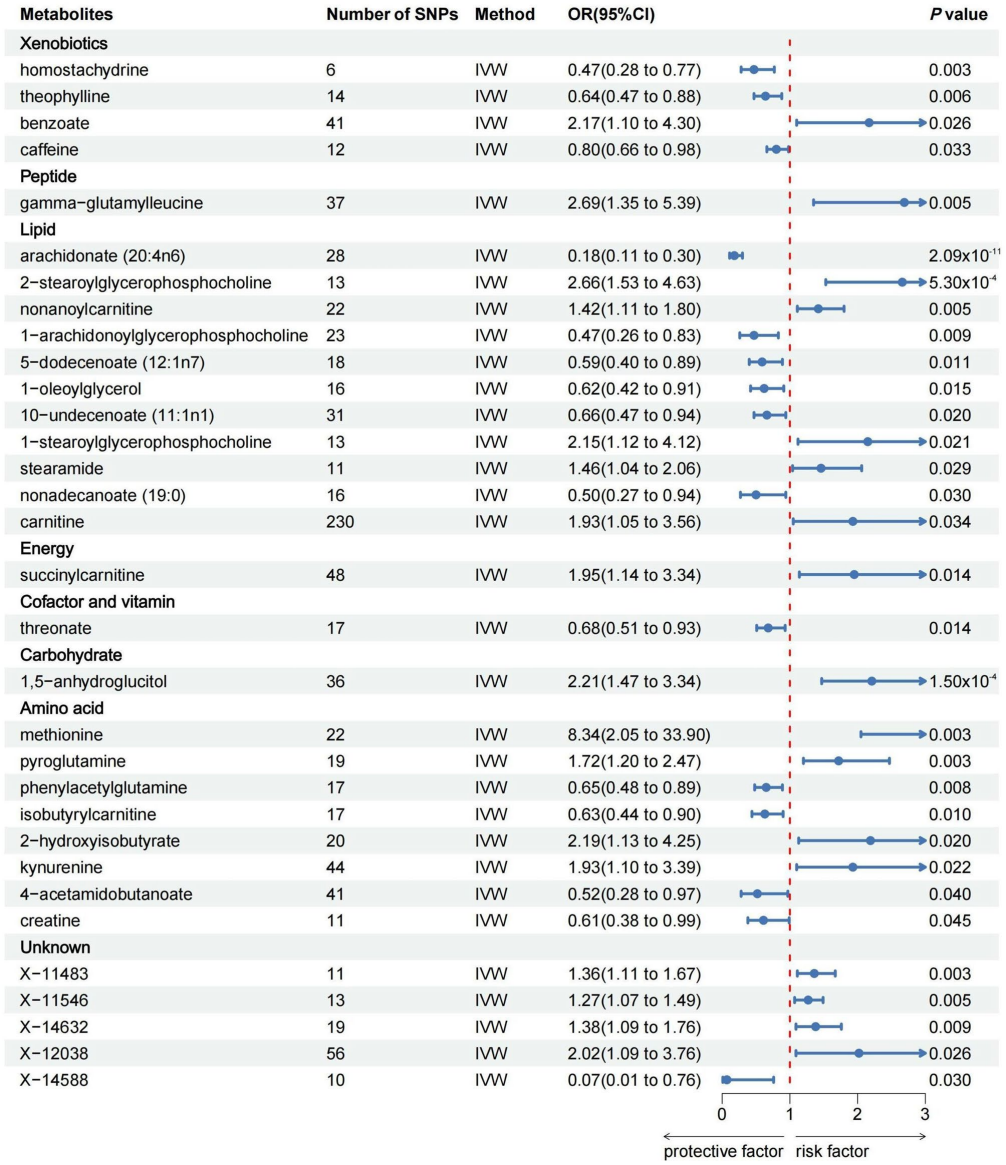
Mendelian randomization results for blood metabolites and the risks of UC based on the inverse variance weighted (IVW) method.

As shown in Fig. 3, 32 metabolites associated with UC were detected by the IVW method (*p* < 0.05), including 11 lipids, 8 amino acids, 4 xenobiotics, 1 carbohydrate, 1 peptide, 1 energy, 1 cofactor and vitamin, and 5 unknown metabolites. Among the known metabolites, 13 metabolites were positively correlated with UC and remaining 14 metabolites were associated with a reduced risk of UC. After FDR correction, only arachidonate (20:4n6) (FDR_ivw_ = 1.01 × 10^–8^), 2-stearoylglycerophosphocholine (FDR_ivw_ = 0.086), and 1,5-anhydroglucitol (FDR_ivw_ = 0.036) remained significant. Regrettably, we did not find metabolites with significant causal effect on both CD and UC.

### Complementary and sensitivity analyses

**Figure 4:**
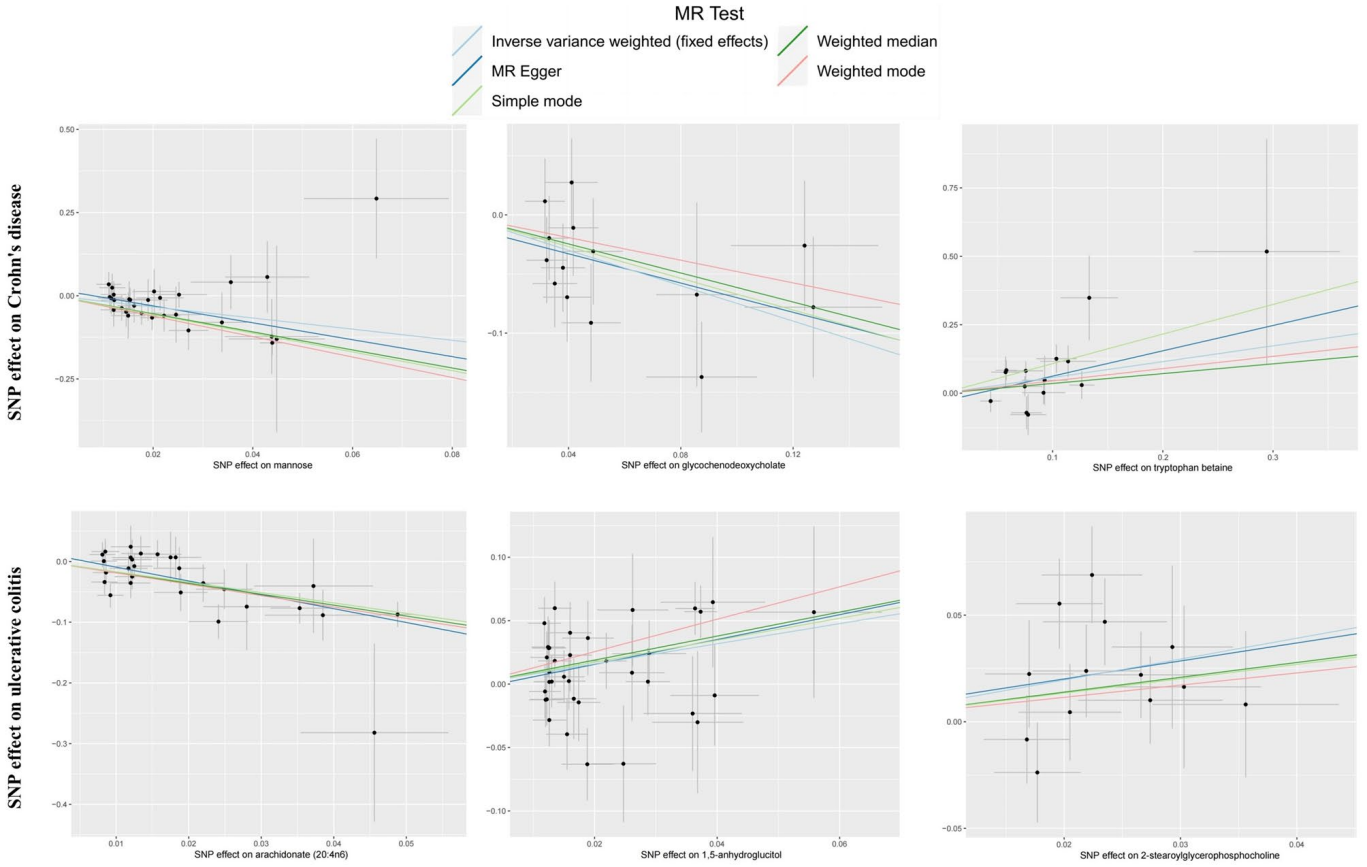
Scatterplot of significantly associated (FDR_ivw_ < 0.1) and directionally consistent estimates between blood metabolites and IBD.

Although the IVW method is highly effective in inferring causal relationships between exposures and disease outcomes, it is known to be susceptible to weak instrument bias. Therefore, we further performed complementary and sensitivity analyses to assess the robustness of causality. As shown in Fig. 4 and Table 1, we performed complementary analyses of meaningful associations obtained by the IVW method with the use of MR-Egger, weighted median, weighted mode, and simple mode methods. The results showed that the IVW method estimates were consistent with the direction and magnitude of MR-Egger, weighted median, weighted mode, and simple mode method estimates, suggesting that the causal relationship is reliable. As shown in Table 2, the results of Cochran Q test showed no heterogeneity. MR-Egger regression and global test of MR-PRESSO suggested that there was no horizontal pleiotropy in all associations. Moreover, the IVW method estimates are in the same direction as the MR-PRESSO method (Supplementary Table S4). In addition, leave-one-out analysis results supported that single SNP did not lead to MR estimation bias (Supplementary Fig. S1). In summary, after combining complementary and sensitivity analyses, we identified 6 metabolites (3 for CD and 3 for UC) that met stringent screening criteria.

**Table 1:** Complementary analyses for causality from blood metabolites on IBD.

Metabolites	Disease	MR analysis		
		Methods	OR (95% CI)	*P*
Mannose	CD	ME	0.08 (0.01–0.53)	0.015
		WM	0.07 (0.02–0.23)	1.42 × 10^–5^
		W	0.05 (0.01–0.21)	4.74 × 10^–4^
		S	0.06 (0.00–0.82)	0.044
Glycochenodeoxycholate	CD	ME	0.54 (0.23–1.24)	0.172
		WM	0.54 (0.30–0.98)	0.043
		W	0.62 (0.31–1.25)	0.202
		S	0.51 (0.18–1.45)	0.228
Tryptophan betaine	CD	ME	2.52 (0.61–10.38)	0.225
		WM	1.43 (0.87–2.36)	0.162
		W	1.57 (0.90–2.74)	0.140
		S	2.95 (1.21–7.16)	0.033
Arachidonate (20:4n6)	UC	ME	0.10 (0.04–0.25)	3.37 × 10^–5^
		WM	0.17 (0.08–0.35)	2.45 × 10^–6^
		W	0.15 (0.07–0.33)	5.49 × 10^–5^
		S	0.18 (0.04–0.76)	0.027
1,5-anhydroglucitol	UC	ME	2.66 (0.88–8.02)	0.091
		WM	2.57 (1.42–4.66)	0.002
		W	3.59 (1.66–7.77)	0.003
		S	2.37 (0.68–8.26)	0.183
2-stearoylglycerophosphocholine	UC	ME	2.32 (0.09–57.08)	0.616
		WM	2.00 (0.89–4.52)	0.094
		W	1.77 (0.61–5.17)	0.315
		S	1.96 (0.55–6.94)	0.318

*ME* MR-Egger, *WM* weighted median, *W* weighted mode, *S* simple mode.

**Table 2:** Sensitivity analyses for causality from blood metabolites on IBD.

Metabolites	Disease	Heterogeneity		MR-Egger		MR-PRESSO	
		Q	*P*	Intercept	*P*	Outliers	Gt *P*
Mannose	CD	21.81	0.79	0.020	0.33	–	0.697
Glycochenodeoxycholate	CD	11.08	0.60	-0.008	0.73	–	0.622
Tryptophan betaine	CD	20.69	0.08	-0.030	0.62	rs2255908	0.112
Arachidonate (20:4n6)	UC	28.02	0.41	0.013	0.14	rs532098, rs7169869	0.507
1,5-anhydroglucitol	UC	45.46	0.11	-0.004	0.72	–	0.127
2-stearoylglycerophosphocholine	UC	15.09	0.24	0.003	0.93	–	0.284

*Gt P* Global test *P.*

### Replication and meta-analysis

**Figure 5:**
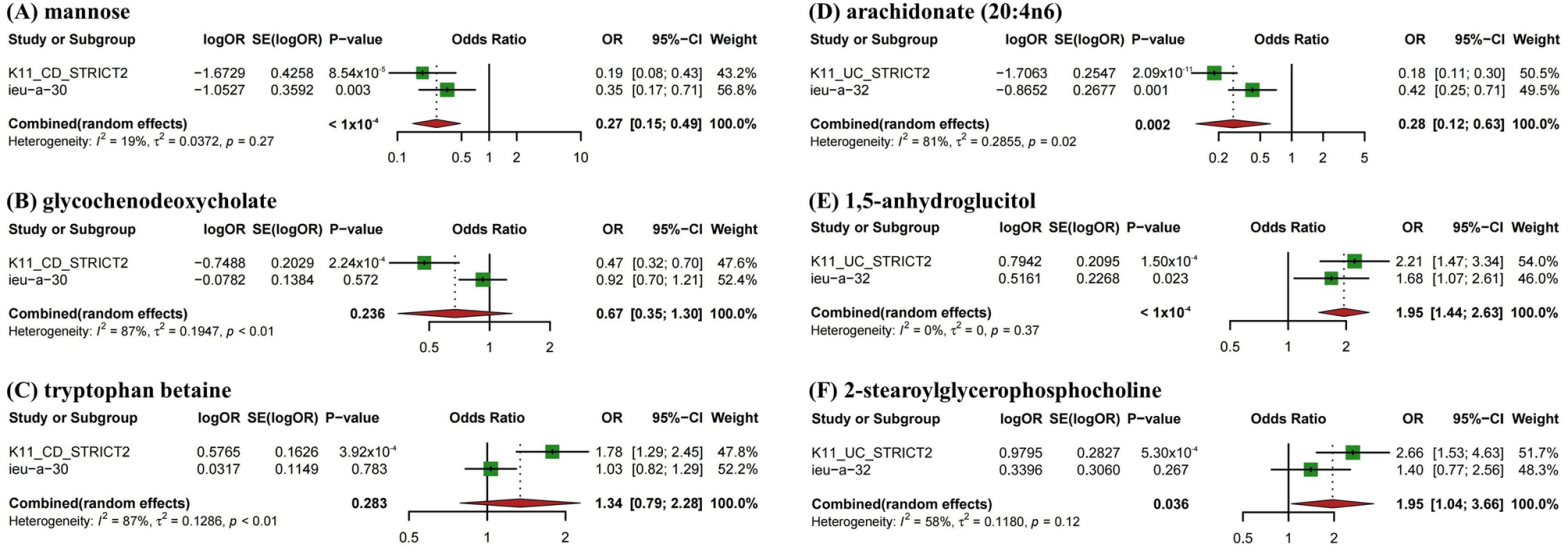
Meta-analysis of the causal associations between metabolites and IBD.

To increase the persuasiveness of the estimates, we performed replication analysis using another IBD GWAS data to further validate our results. Combining the 2 GWAS datasets, a meta-analysis was performed for 6 metabolites that met stringent screening criteria. The results were as expected, and similar trends were observed for these 6 metabolites in another GWAS data (Fig. 5). In CD, in detail, genetic susceptibility for higher levels of mannose (OR = 0.27, 95% CI 0.15–0.49, *P* < 1 × 10^–4^) decreased risk of CD. The glycochenodeoxycholate (*P* = 0.236) and tryptophan betaine (*P* = 0.283) were not observed to be statistically significant, although consistent directions were shown in both MR analyses. Therefore, we discarded the glycochenodeoxycholate and tryptophan betaine. In UC, genetic susceptibility for higher levels of arachidonate (20:4n6) (OR = 0.28, 95% CI 0.12–0.63, *P* = 0.002) decreased risk of UC, while genetic liability for higher levels of 1,5-anhydroglucitol (OR = 1.95, 95% CI 1.44–2.63, *P* < 1 × 10^–4^), 2-stearoylglycerophosphocholine (OR = 1.95, 95% CI 1.04–3.66, *P* = 0.036) promoted susceptibility to UC.

### Genetic correlation and direction validation

The results of the LDSC analysis showed weak evidence of a genetic correlation between 4 metabolites and IBD. Rg ranges from -0.8046 to 0.0682, the standard error from 0.0425 to 0.4230, and the p-value from 0.0527 to 0.9599 (*p* > 0.05). These results suggested that these shared genetic components did not confound the MR estimates (Supplementary Table S5). Furthermore, we further performed the Steiger test to examine whether there was reverse causality between metabolites and IBD. The results of Steiger do not support the existence of reverse causal effects between metabolites and IBD (Supplementary Table S6).

### Metabolic pathway analysis

Based on the metabolites that passed the threshold of association by IVW (P_IVW_ < 0.05), the metabolic pathway analysis identified 4 significant metabolic pathways in IBD (Supplementary Table S7). Specifically, our results show that the phenylalanine metabolism (*P* = 0.051) and ether lipid metabolism (*P* = 0.099) pathways were found to be associated with the pathogenetic process of CD, whereas arginine and proline metabolism (*P* = 0.008) and caffeine metabolism (*P* = 0.038) were considered to be associated with UC.

## Discussion

In this study, we conducted a two-sample MR analysis to causally assess 486 blood metabolites and the risk of IBD through a rigorous MR design. We performed initial IVW analyses and identified a total of 63 causal relationships involving 59 metabolites and two IBD subtypes. Next, we performed complementary and sensitivity analyses on these metabolites. Then, to further ensure the reliability and stability of the results, we performed replication, meta-analysis, Steiger test, and LDSC. Ultimately, we identified four significant causal associations between four metabolites and two IBD subtypes. In addition, we detected four significant metabolic pathways involving two IBD subtypes. To our knowledge, this is the first MR study to systematically and comprehensively assess the causal role of human blood metabolites in IBD. Our study provides new insights into the role of gene-environment interactions in the pathogenesis of IBD and offers potential inspiration for further precise diagnosis and treatment.

In recent decades, IBD has been increasingly recognized as a metabolism-related disease, not only because it can coexist with a variety of metabolic disorders, but also because metabolites and metabolic pathways associated with IBD continue to be identified in metabolomic studies. Blood, feces, and synovial fluid are typical sample sources for metabolomics identification^43^. Among them, blood is considered a good source because it contains a large number of detectable metabolites and can be easily obtained in large samples, thus helping to screen for circulating markers of IBD risk. Moreover, blood metabolites provide a visual snapshot of biological mechanisms as they capture both endogenous and exogenous processes^12,44^. Metabolomics studies using serum/plasma have reportedly found altered metabolic profiles in IBD patients, and the most common metabolites are lipids, amino acids, and carbohydrates^11,45,46^. Despite the increasing number of studies on blood metabolism of IBD, there are still a lack of comprehensive and systematic studies to assess the causal relationship between IBD and blood metabolites, and thus the contribution to the precise diagnosis and treatment of IBD is limited. Therefore, we conducted a pivotal MR study with the aim of elucidating the causal relationship between blood metabolites and IBD and the metabolic pathways they may be involved in, thereby providing a reference direction for colorectal cancer screening and treatment.

Our findings suggested that genetic sensitivity to high levels of mannose can keep the body safe from CD. Mannose is a naturally occurring biologically active monosaccharide involved in glycoprotein metabolism and synthesis with anti-inflammatory and antioxidant activities. Metabolomics analyses showed that serum mannose concentrations were elevated in patients with CD, reflecting possible defects in the uptake, transport, or catabolism of endogenous mannose in cells^47^. A recent study showed that mannose could prevent endoplasmic reticulum stress induced by macrophage-secreted tumor necrosis factor-alpha (TNF-α) in intestinal epithelial cells by normalizing protein N-glycosylation, thus maintaining epithelial integrity. Moreover, mannose directly inhibited the production of TNF-α from macrophages by lowering the level of glyceraldehyde 3-phosphate and attenuates intestinal inflammation^48^. Another recent study found that mannose treatment attenuated intestinal barrier damage in IBD mouse models. The underlying mechanism may be that mannose limits cathepsin B release by enhancing lysosomal integrity, thereby preventing mitochondrial dysfunction and myosin light chainkinase (MLCK)-induced disruption of tight junctions in the context of intestinal epithelial injury. Notably, their experimental data demonstrated that mannose combined with mesalamine had a synergistic therapeutic effect on colonic inflammation in mice^49^. However, the direct mechanism of action of mannose in the treatment of IBD is unclear. Our results further support these findings and emphasize the importance of mannose as a protective factor in CD progression.

Arachidonate (20:4n6) is one of the polyunsaturated fatty acids found in cell membranes and is essential for maintaining cellular functions. Arachidonate can act as the substrate for the generation of a series of metabolic derivatives such as prostaglandins, thromboxanes, leukotrienes in response to cyclooxygenase, lipoxygenases, and cytochrome P450 epoxygenation. Metabolomics analysis showed that serum arachidonate levels were reduced in UC patients compared to healthy controls^50^. The role of arachidonate in UC remains incompletely elucidated. Several previous studies have shown that in mouse models of colitis, prostaglandin E2, a metabolic derivative of arachidonate, can exacerbate intestinal inflammation by increasing inflammatory cytokine secretion through promoting Th1 cell differentiation and Th17 cell proliferation^51,52^. Moreover, arachidonate can be metabolized to produce heparin A3 (HXA3), which promotes neutrophil migration into the lumen^53^. However, as studies progressed, some found that arachidonate could inhibit intestinal inflammation through the production of prostaglandins D2, poxyeicosatrienoic acids, 15-hydroxy eicosapentaenoic acid^54–56^. Notably, due to the complexity of the Arachidonate metabolic network, there are no convincing in vivo experimental data confirming a causal role between blood Arachidonate levels and UC. Our findings suggest that genetic susceptibility to high levels of Arachidonate plays a protective role in the development of UC, which needs to be further explored in in vivo experimental models.

We also confirmed genetic predisposition to higher levels of 1,5-anhydroglucitol and 2-stearoylglycerophosphocholine were detrimental to UC. To date, research on the relevance of 1,5-anhydroglucitol to UC is minimal. In patients with Glycogen Storage Disease type 1b (GSDIb), the accumulation of 1,5-anhydroglucitol-6-phosphate, which is produced by the metabolism of 1,5-anhydroglucitol in neutrophils, leads to neutropenia and neutrophil dysfunction^57,58^. Several recent studies have shown that the use of dapagliflozin or empagliflozin can induce IBD remission in patients with GSDIb by lowering plasma concentrations of 1,5-anhydroglucitol^59,60^. Therefore, it is reasonable to hypothesize that 1,5-anhydroglucitol can contribute to the development of UC by impairing neutrophil function. In contrast, reports on 2-stearoylglycerophosphocholine are extremely limited, with a few studies suggesting that 2-stearoylglycerophosphocholine may promote the development of severe COVID-19^61^. The causal role of 2-stearoylglycerophosphocholine on UC needs to be further explored.

Furthermore, in this study, we identified 4 metabolic pathways associated with the development of IBD, some of which are well documented in experimental studies for their role in the pathogenesis of IBD. Arginine is a semi-essential amino acid that participates in the urea cycle and arginine/proline metabolism. In humans, arginine is metabolized mainly by the arginase pathway to produce urea and ornithine; and by the nitric oxide synthase (NOS) pathway to produce citrulline and nitric oxide (NO)^62^. It is reported that NO has a protective role in colitis^63^. Moreover, ornithine is converted to proline by ornithine transaminase, which promotes the repair of the intestinal barrier during inflammation^64^. These data suggest that enhanced arginine and proline metabolism may be protective against UC. Caffeine is a natural chemical with stimulant effects. Previous studies have shown that caffeine can reduce the risk of UC and ameliorates colitis^65,66^. However, there are few experimental studies on the role of ether lipid metabolism and phenylalanine metabolism in the pathogenesis of IBD, which warrant further experimental exploration.

The present study has several strengths. First, the most significant strength of this study is that we covered a large number of genetic variables to analyze the relationship between blood metabolites and IBD. A total of 486 metabolites were covered in this study, which is of significant clinical research value. Second, rigorous MR analysis was used to discard unavoidable pitfalls of previous studies, such as confounding interference and reverse causality. In order to produce convincing estimates, a series of methods were implemented to ensure that violations of MR assumptions were eliminated to the greatest extent possible, according to the STROBE-MR Statement^15^. Specifically, the consistency of the five MR method estimates in direction and magnitude ensured the robustness of the results. At the same time, sensitivity analyses were conducted to exclude the effects of heterogeneity and pleiotropy on the estimates. Third, the reliability of the results was further validated by additional GWAS data used for repeated analyses and meta-analysis. Finally, we used LDSC to assess the genetic correlation between metabolites and IBD, which made the MR estimates more convincing.

There are some limitations to our study. First, all metabolite GWAS and IBD GWAS data were from European populations. Although this largely avoids population heterogeneity, MR results should be further validated in other populations to verify their generalizability with more GWAS data in future studies. Next, the number of metabolite-associated SNPs was limited. To address this issue, we set a slightly relaxed threshold for MR analysis, which is a common practice in other studies. Moreover, although we used PhenoScanner V2 to exclude IVs associated with confounders (such as depression, being a worrier, smoking, taking contraceptives, having sclerosing cholangitis), it was a limitation that numerous other IBD factors could not be fully accounted for. Finally, this study covers a relatively comprehensive metabolite profile, but the functions and mechanisms of some metabolites in the disease are not yet fully understood, which limits our interpretation of the results of this MR analysis. Our findings need to be validated by rigorous RCTs.

## Conclusion

In conclusion, we identified four robust causal associations between four metabolites and two IBD subtypes. Through metabolic pathway analysis, four important metabolic pathways associated with IBD were identified. The finding of our study suggested that the identified metabolites and metabolic pathways can be considered as useful circulating metabolic biomarkers for the screening and prevention of IBD in clinical practice, as well as candidate molecules for future mechanism exploration and drug target selection.

### Supplementary Information

Supplementary Figure 1.

Supplementary Tables.

## Data Availability

The datasets presented in this study can be found in online repositories. The names of the repository/repositories and accession number(s) can be found in the article/Supplementary Material.
