# Supplementary material for: Causality of genetically determined blood metabolites on inflammatory bowel disease: a two-sample Mendelian randomization study
Source: Sci Rep. 2024 Jul 16;14:16361. doi: 10.1038/s41598-024-67376-0 (PMC11252329; doi:10.1038/s41598-024-67376-0)

**Supplementary Figure**

Supplementary Figure 1: Forest plots for the Mendelian randomization (MR) leave-one-out analysis of the significant inverse variance weighted estimates.

Supplementary Figure 1: Forest plots for the Mendelian randomization (MR) leave-one-out analysis of the significant inverse variance weighted estimates (FDR_ivw_<0.1).


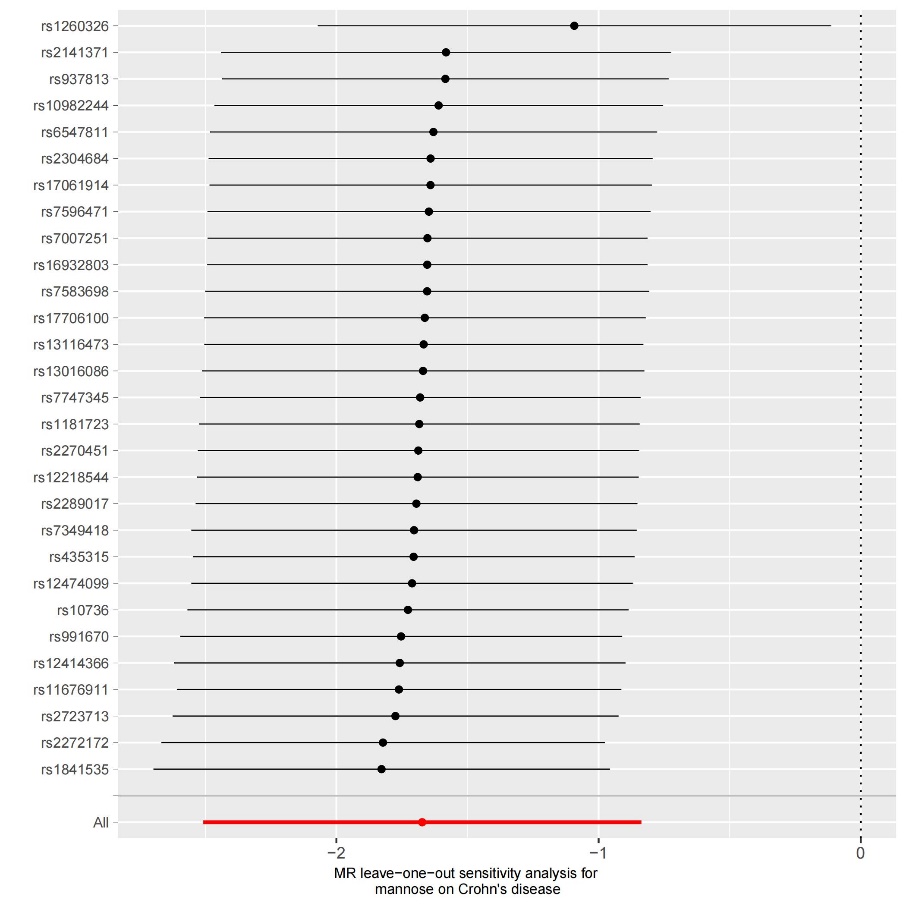


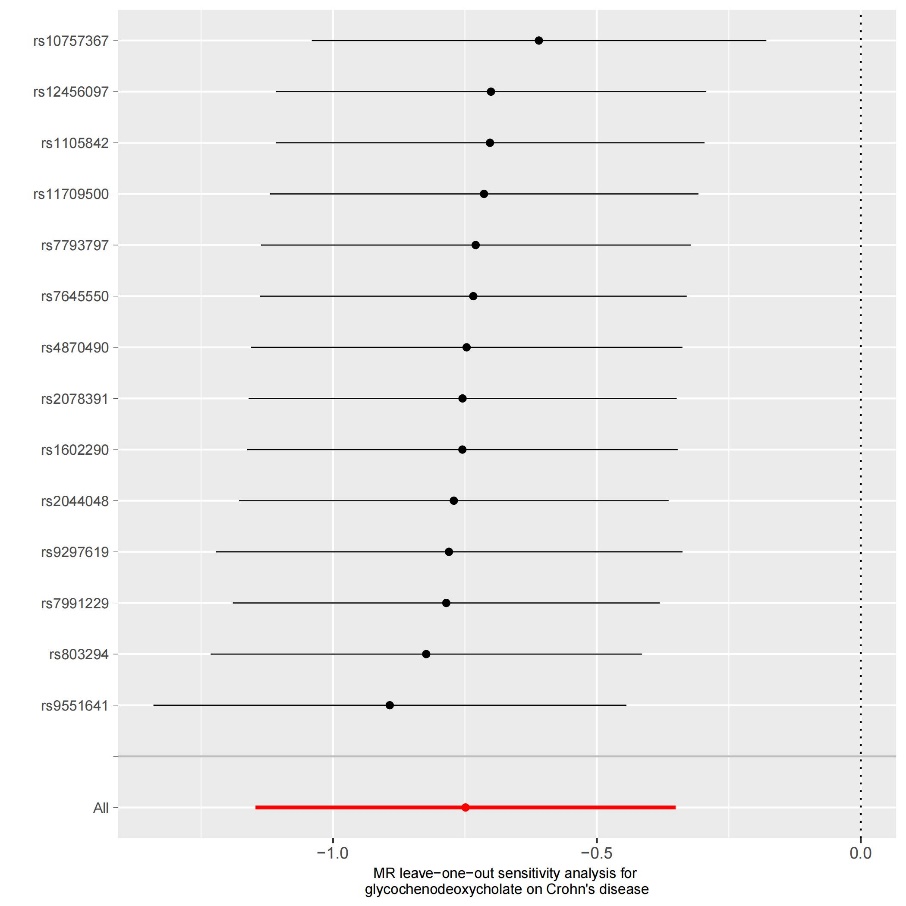


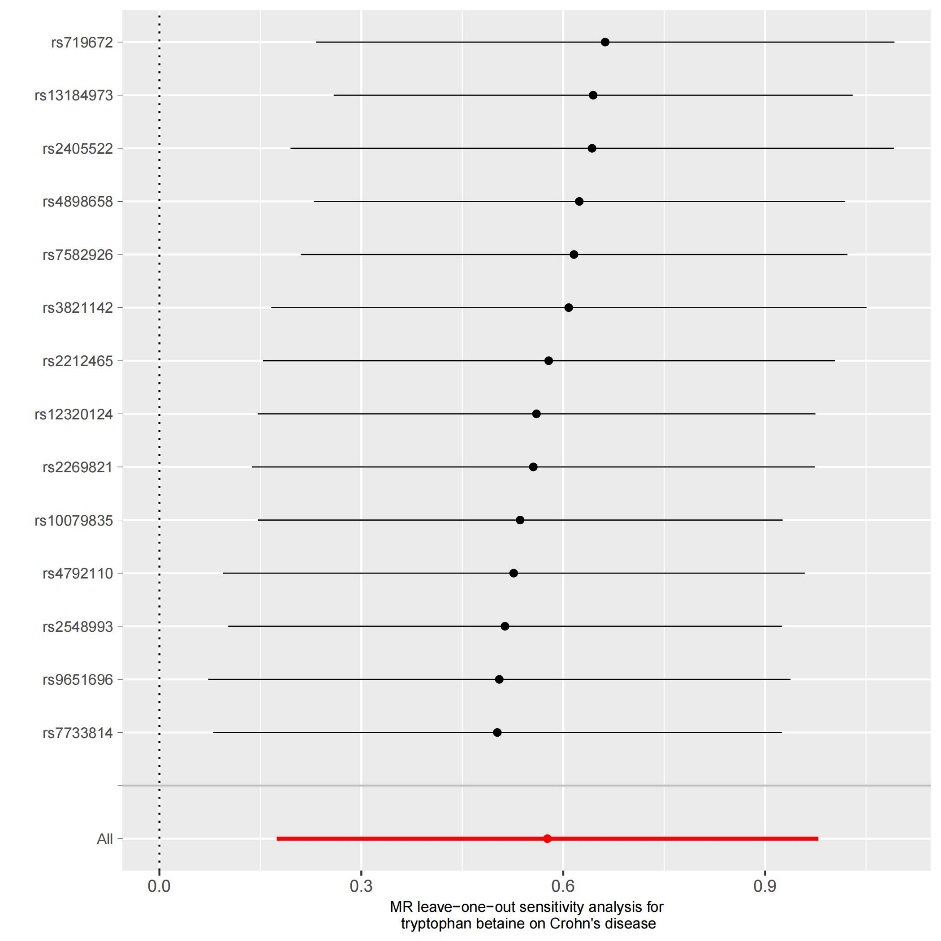


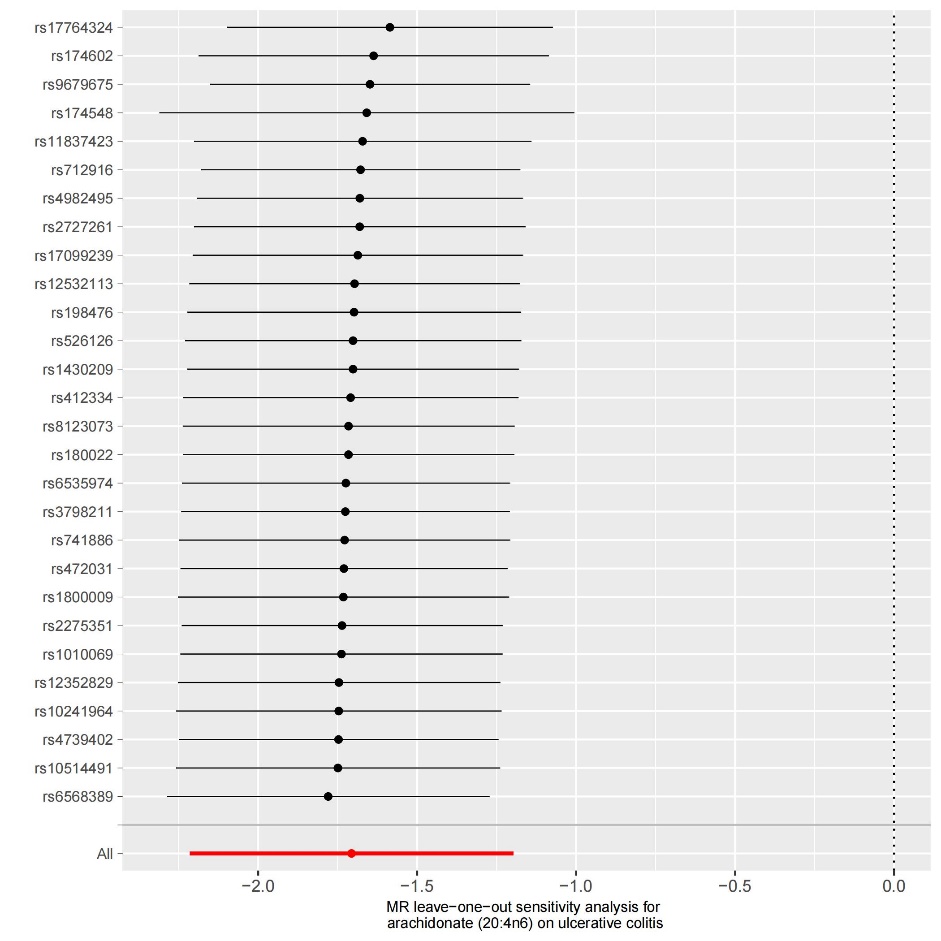


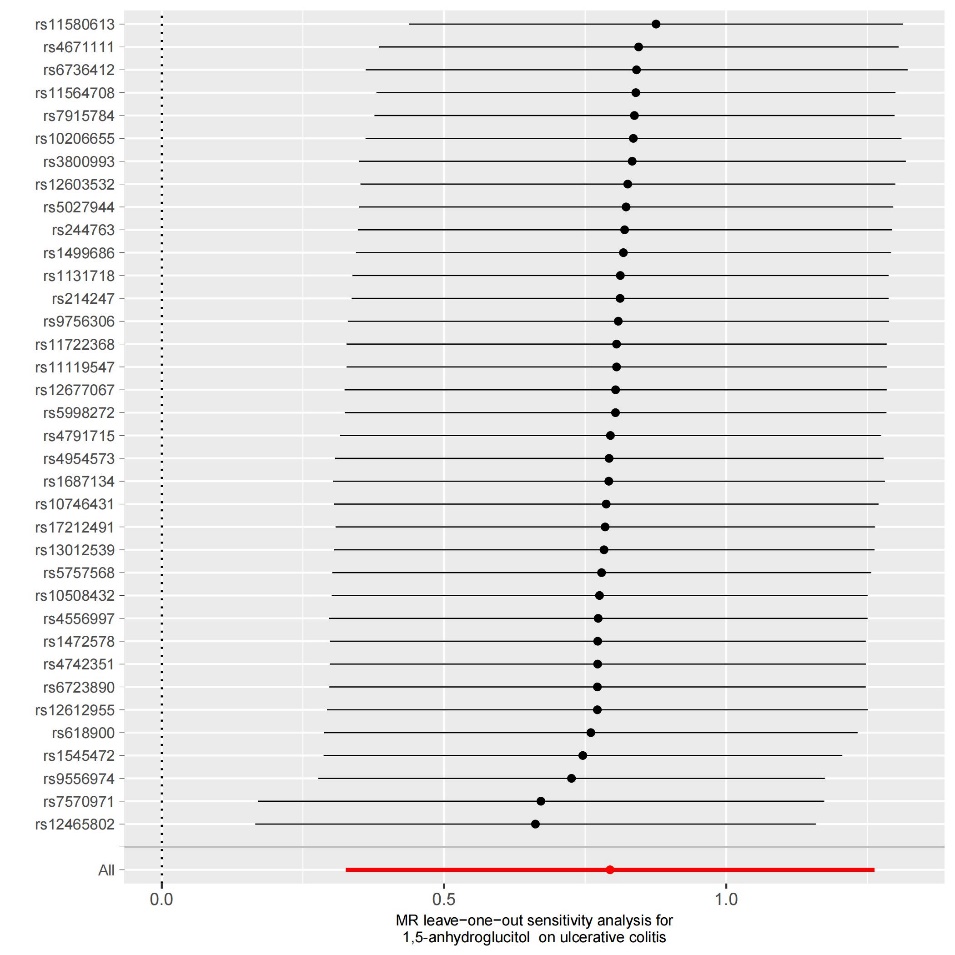


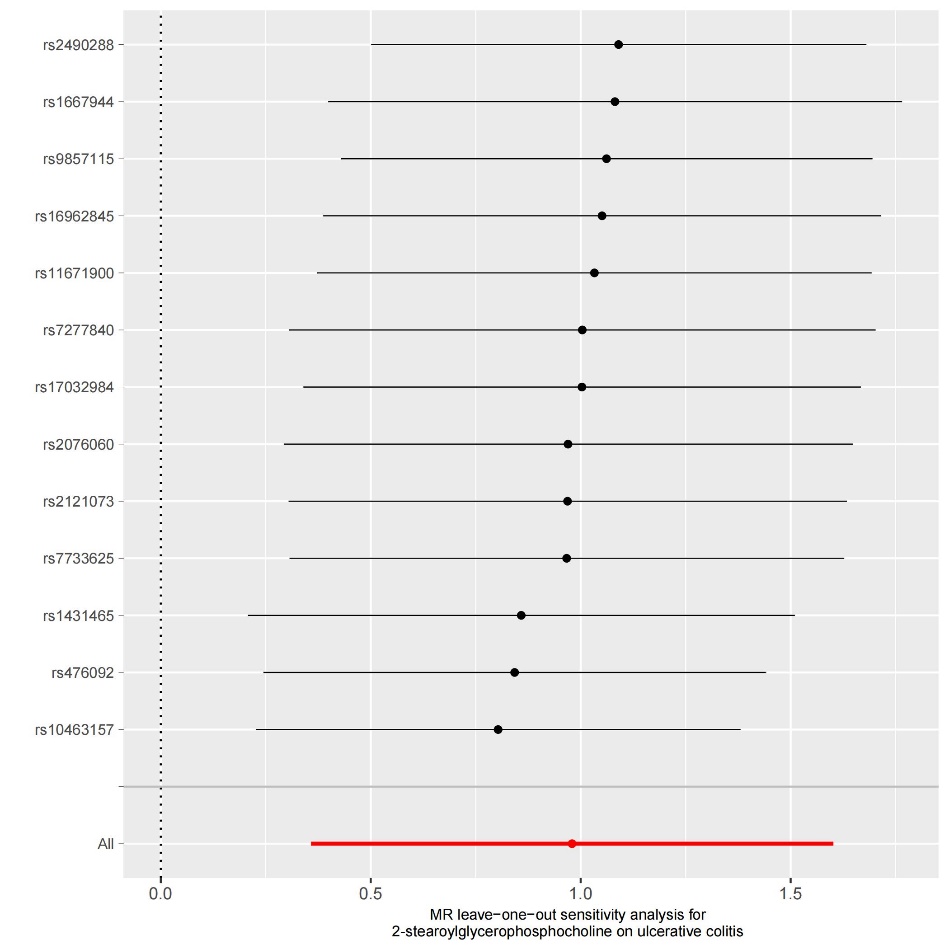

Supplement: Supplementary file 1 — Supplementary Figure 1. [file 41598_2024_67376_MOESM1_ESM.docx]
